# Engineering a conduction-consistent cardiac patch with rGO/PLCL electrospun nanofibrous membranes and human iPSC-derived cardiomyocytes

**DOI:** 10.3389/fbioe.2023.1094397

**Published:** 2023-02-08

**Authors:** Yao Tan, Ying Chen, Tingting Lu, Nevin Witman, Bingqian Yan, Yiqi Gong, Xuefeng Ai, Li Yang, Minglu Liu, Runjiao Luo, Huijing Wang, Stefano Ministrini, Wei Dong, Wei Wang, Wei Fu

**Affiliations:** ^1^ Institute of Pediatric Translational Medicine, Shanghai Children’s Medical Center, School of Medicine, Shanghai Jiao Tong University, Shanghai, China; ^2^ Department of Clinical Neuroscience, Karolinska Institute, Stockholm, Sweden; ^3^ Department of Pediatric Cardiothoracic Surgery, Shanghai Children’s Medical Center, School of Medicine, Shanghai Jiao Tong University, Shanghai, China; ^4^ Department of Anesthesiology, Fudan University Shanghai Cancer Center, Shanghai, China; ^5^ Department of Oncology, Shanghai Medical College, Fudan University, Shanghai, China; ^6^ Center for Molecular Cardiology, University of Zurich, Zurich, Switzerland; ^7^ Department of Medicine and Surgery, Internal Medicine, Angiology and Atherosclerosis, University of Perugia, Perugia, Italy; ^8^ Shanghai Key Laboratory of Tissue Engineering, Shanghai 9th People’s Hospital, School of Medicine, Shanghai Jiao Tong University, Shanghai, China

**Keywords:** cardiac patch, induced pluripotent stem cells, reduced graphene oxide, conduction consistency, electrospun nanofibrous

## Abstract

The healthy human heart has special directional arrangement of cardiomyocytes and a unique electrical conduction system, which is critical for the maintenance of effective contractions. The precise arrangement of cardiomyocytes (CMs) along with conduction consistency between CMs is essential for enhancing the physiological accuracy of *in vitro* cardiac model systems. Here, we prepared aligned electrospun rGO/PLCL membranes using electrospinning technology to mimic the natural heart structure. The physical, chemical and biocompatible properties of the membranes were rigorously tested. We next assembled human induced pluripotent stem cell-derived cardiomyocytes (hiPSC-CMs) on electrospun rGO/PLCL membranes in order to construct a myocardial muscle patch. The conduction consistency of cardiomyocytes on the patches were carefully recorded. We found that cells cultivated on the electrospun rGO/PLCL fibers presented with an ordered and arranged structure, excellent mechanical properties, oxidation resistance and effective guidance. The addition of rGO was found to be beneficial for the maturation and synchronous electrical conductivity of hiPSC-CMs within the cardiac patch. This study verified the possibility of using conduction-consistent cardiac patches to enhance drug screening and disease modeling applications. Implementation of such a system could one day lead to *in vivo* cardiac repair applications.

## 1 Introduction

The natural heart has a special directional arrangement of cardiomyocytes (CMs) and a unique electrical conduction system that is critical for maintaining effective contraction (Nunes et al., 2013; Ruan et al., 2016; Ronaldson-Bouchard et al., 2018). The design of a cardiac muscle patch with well-aligned cardiomyocytes and improved conduction consistency is required for higher throughput applications. Of interest, several studies have been conducted revealing how to orient cardiovascular cell-types into stable arrangements (Pijnappels et al., 2008; Tulloch et al., 2011; Veldhuizen et al., 2020). Electrospinning technology is one of the most widely used technologies that uses polymer solutions or melts to spray spin fibers in a strong electric field (Kitsara et al., 2017; Xue et al., 2019). The many advantages of this technology include a simple manufacturing device, low spinning costs, various spinnable materials and controllable processes (Shi et al., 2022). Through specific methods, fiber filaments can be induced by a specific arrangement that has a certain contact guidance function for tissue cell arrangement. Among the various materials used for electrospinning, poly(L-lactide-co-ε-caprolactone) (PLCL) exhibits high elasticity, a controllable degradation rate, and low cytotoxicity, making it a good choice for tissue-engineering membrane materials (Jin et al., 2009). The biocompatibility and mechanical strength of PLCL nominate the membrane material to be used for myocardial patches (Jung et al., 2008; Prabhakaran et al., 2009; Kuang et al., 2020). Because of the poor electrical conductivity of PLCL, it is necessary to explore options for enhancing this property through additive conductive materials.

Reduced graphene oxide (rGO) is a derivative of graphene that exhibits unparalleled mechanical characteristics, excellent conductivity and a large surface area that can be chemically functionalized (Yang et al., 2013). rGO can be processed via the removal of oxidation groups, which enhances the conductivity of rGO over that of graphene oxide, yet rGO retains excellent biocompatibility and low toxicity (Tahriri et al., 2019). In addition, rGO has been shown to support a number of beneficial cellular properties including adhesion, proliferation, and differentiation (Jin et al., 2015). rGO has been implemented to modify cellular properties in a number of cell-types and applications ranging from nerves (Guo et al., 2016; Qin et al., 2019; Wang et al., 2019), skin (Jin et al., 2019; Liang et al., 2019; Yan et al., 2019), muscle (Mu et al., 2016; Qiao et al., 2018) and orthopedics (Mehrali et al., 2014; Xiong et al., 2017; Zhou et al., 2019). In cardiac applications, researchers have found primary CMs cultured on composite rGO-GelMA scaffolds exhibited better biological activities such as cell viability, proliferation and maturation compared to those CMs cultured on gelatin methacryloyl hydrogels, demonstrating that rGO as a conductive material can promote CM maturation (Shin et al., 2016; Lee et al., 2019). In addition, rGO combined with poly(ester-amide) promotes the differentiation of mesenchymal stem cells into CMs (Stone et al., 2019). Furthermore, rGO was previously shown to support antibacterial effects (Norahan et al., 2019; Yang et al., 2019), as well as antioxidant effects, the latter of which was shown to effectively clear the reactive oxygen species produced by myocardial injury (Choe et al., 2019). These studies suggest that rGO is an excellent material for engineering myocardial patches.

Human induced pluripotent stem cells (hiPSCs) have become a widely used source of material for disease modeling and therapeutic applications of tissue regeneration (Takahashi and Yamanaka, 2006). Numerous protocols have been employed to generate large numbers of CMs robustly and efficiently from hiPSCs (Laflamme et al., 2007; Burridge et al., 2014; Rahmani et al., 2019). More recently, hiPSC-derived CMs (hiPSC-CMs) have been used for cardiovascular regenerative therapy as a potential therapeutic agent applicable for tissue-engineered myocardial construction. Personalized treatments utilizing hiPSC-CMs have been shown to prevent immune responses (Nishikawa et al., 2008; Nelson et al., 2010), and several studies have verified the safety and practicality of iPSC-CMs in cardiac injury (Nelson et al., 2009; Shiba et al., 2016; Yoshida and Yamanaka, 2017).

**FIGURE 1:**
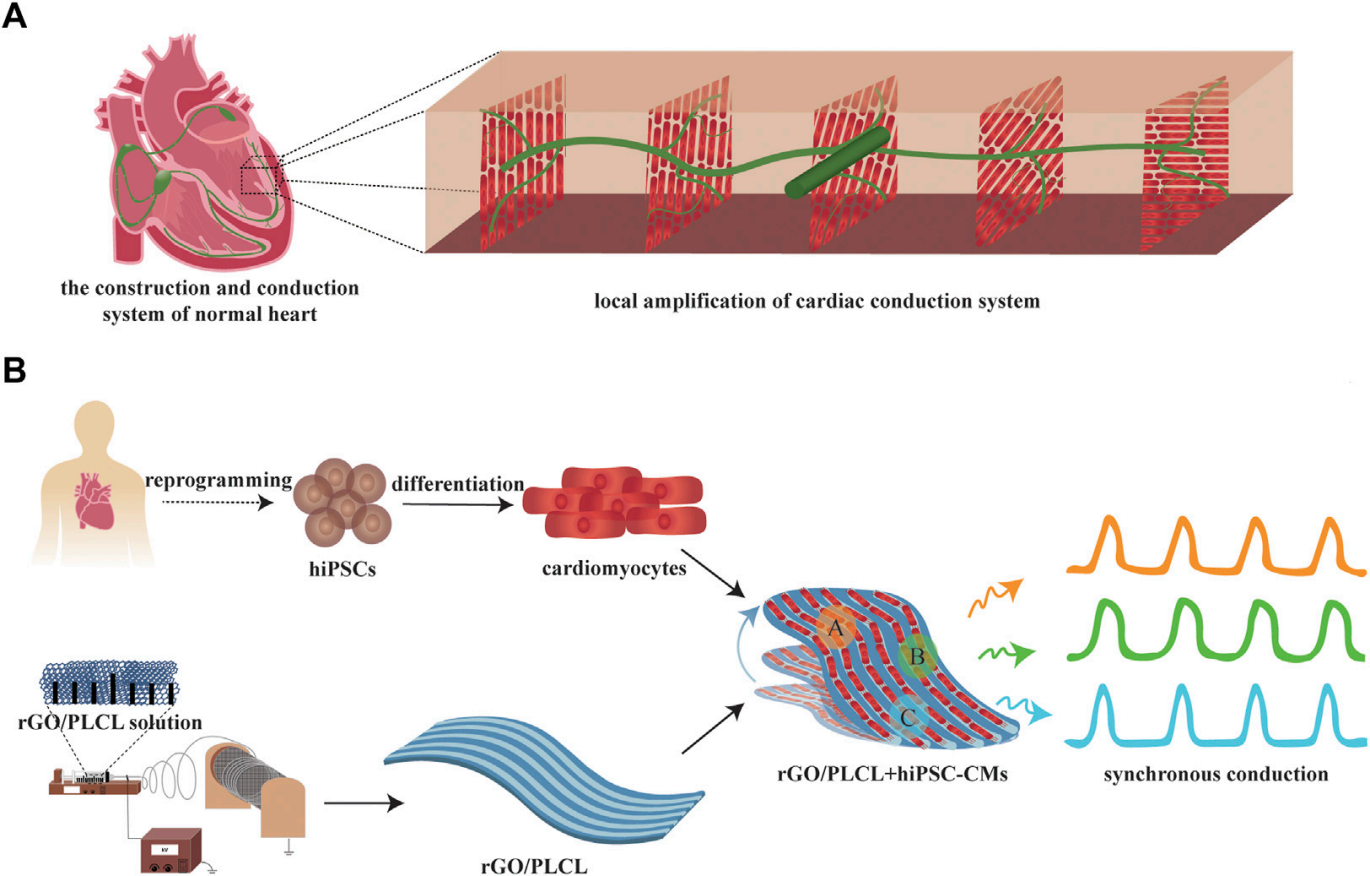
Scheme of the construction of a conduction-consistent cardiac patch. **(A)** Scheme of the construction and conduction system of normal heart; **(B)** Scheme of the construction and detection of the conduction-consistent cardiac patch.

In this study, we first prepared aligned electrospun rGO/PLCL membranes using electrospinning technology and tested their mechanical properties, electrical conductivities, and biocompatibilities. Thereafter, we combined the hiPSC-CMs and aligned electrospun rGO/PLCL membranes to construct a myocardial patch and tested the conduction consistency of this patch. In summary, we designed and manufactured a myocardial muscle patch that can simulate the structure and electrophysiological characteristics of normal cardiac tissue *in vitro* (Figure 1). The novel myocardial patch described herein offers excellent application prospects for developmental cardiac studies, drug screening applications, and *in vitro* disease modeling at low cost.

## 2 Materials and methods

### 2.1 Preparation of rGO/PLCL electrospun membranes

rGO (Changzhou, China) is reduced graphene oxide by chemical reduction. rGO solutions with different concentrations were prepared by dispersing rGO in hexafluoroisopropanol (HFIP, J&K Scientific, Beijing, China). 7 (w/v) % PLCL (Daigang Bioengineering, Jinan, China) was stirred and dissolved with HFIP. Finally, rGO/PLCL solutions with final rGO concentrations of 0, 0.1 wt%, 0.5 wt%, 1 wt%, 2 wt%, 4 wt%, 8 wt% were prepared. These solutions were stirred at room temperature for 48 h. 10 KV high voltage was used for electrospinnig (Teslaman, Dalian, China). The distance from needle tip (14 G) to the collector was 9 cm, and the flow rate was 1.5 mL/hr. The collector speed was 1000 rpm/min. The temperature and humidity were controlled at 20°C–25°C and 30%–40% respectively. The anisotropic structure was obtained by installing 400 mesh wire mesh on the electrospinning shaft, which was donated by Professor Zhang of Shanghai Children’s Medical Center. The addition of rotating collector wire mesh can help to obtain directionally arranged fibers (Chen et al., 2020; Wang et al., 2020; Yao et al., 2023).

### 2.2 Characterization of the membranes

The characterization of surface morphology was performed by a scanning electron microscope (SEM, TESCAN, Shanghai, China). Diameters of nanofibers were determined with ImageJ software by randomly selecting 200 data points. The fiber angle was measured by Photoshop and analyzed by ImageJ. The surface characterization was carried out by Fourier transform infrared spectroscopy (FTIR, ThermoFisher Scientific, Waltham, United States) and X-ray diffraction (XRD, Brukeur, Karlsruhe, Germany). The FTIR is processed by κBr method over a wavenumber range of 500–4,000 cm−1. XRD working parameters: voltage 40 KV, current 30 mA, step size 0.02. The surface wettability was determined by the Water Contact Angle (Kruss, Hamburg, Germany). Droplets of 12 μL were placed on the surface of membranes.

### 2.3 Mechanical testing

The membranes were cut into 30 mm*10 mm rectangle. The range of thickness was 0.3 mm–0.4 mm. The Table type Uniaxial Tensile Testing Machine (INSTRON, Boston, USA) was used to test the mechanical properties of the membranes in dry and hydrated states. For hydrated states, the membranes were soaked in water for 10 min prior to testing. After fixing the material on the instrument, stress-strain curves were recorded at the speed of 10 mm/min. Young’s modulus, final stress and deformation at break point was determined using the stress-strain curve. Three samples were tested for each group.

### 2.4 Electrical resistivity

The electrical resistivity of the membranes was determined with a four-probe method using Surface Resistance Tester for Insulating and Conducting Electrostatic Materials (Suzhou Jingge Electronic Co., Ltd., China, Suzhou, China) with its correction coefficient of 23.76.

### 2.5 Antioxidant test

The membranes used for antioxidant test weighed 4.5 mg, which was determined according to the standard curve of the anti-oxidation kit. We ensure that the OD value of each sample is within the range of the standard curve to ensure the reliability of the experimental data. Total antioxidant activity was determined using Total Antioxidant Capacity Assay Kit with a Rapid ABTS method following the manufacturer’s instruction. Briefly, 20 μL of peroxidase solution and 170 μL of ABST working solution was added to 96-well plate which was in contact with the membrane fragments. The absorbance at 405 nm was read with a microplate reader following 6 min of incubation (Thermo, Massachusetts, USA). The relative antioxidant capacity of these membranes was obtained according to the standard curve. Each experiment was repeated 5 times.

### 2.6 Cell culture

3T3 cells kindly provided by Chinese Academy of Sciences, were cultured in a DMEM-F12 medium containing 10% FBS. The cultures were maintained in a humidified incubator in constant temperature (37°C) and humidity (5% CO2), and underwent medium exchange every 3 days. 2*10^5^ of 3T3 were cultivated on the membranes.

The human iPSC cell line (DYR0100) was kindly provided by Stem Cell Bank, Chinese Academy of Sciences. hiPSCs culture and differentiation were carried out as previously described (Lian et al., 2013). Briefly, hiPSCs were maintained in TeSR-E8 (STEMCELL, Vancouver, CA). hiPSCs were grown in 6-well plates and were passaged when the cells reached 80%–90% confluency using Accutase (STEMCELL, Vancouver, CA). To prepare for differentiation, the cells were collected, centrifuged for 5 min (1,000 rpm at room temperature), and 100 million cells were resuspended with TeSR-E8 culture medium supplemented with 5 μM Y27632 (STEMCELL, Vancouver, CA) into 12-well plates and cultured in incubators with constant temperature (37°C) and humidity, 5% CO2. After 48 h culturing (D0), TeSR-E8 medium was replaced with RPMI1640/B27 minus insulin (Life technologies, Waltham, United States) with 12 μM CH99021 (STEMCELL, Vancouver, CA). Then at 36 h after culture in 12-well plates (D1), the medium was replaced with new RPMI1640/B27 minus insulin. 2 days later (D3), the medium was replaced with RPMI1640/B27 minus insulin supplemented with 5 μM IWP-2 (STEMCELL, Vancouver, CA). Subsequently the culture medium was replaced with RPMI1640/B27 minus insulin 2 days later. On D7, the culture medium was replaced by RPMI1640/B27 with insulin (Life technologies, Waltham, United States). Hereafter fresh culture medium is replaced every 2 days. After D10, spontaneous beating cardiomyocytes can be typically observed on the plate. 5*10^5^ of hiPSC-CMs were cultivated on the membranes.

### 2.7 Confocal fluorescence imaging

Cells seeded on the aligned electrospun membranes were washed with PBS 3 times. The cells were fixed by applying 4% PFA for 30 min. Subsequently, the cells were washed with PBS for 3 times then permeabilized using 0.5% Triton X-100. The cells were again washed 3 times with PBS, followed by the addition of 5% BSA for 2 h at room temperature for blocking. The following primary antibodies were used for immunohistochemistry: Anti-TRA-1-60 (Merck Millipore, Darmstadt, Germany), Anti-OCT-4 (Epitomics, Cambridge, United States), Anti-SOX-2 antibody (Epitomics, Cambridge, United States), Anti-NANOG (Epitomics, Cambridge, United States), Cardiac Troponin-T (Proteintech, Chicago, United States), Anti-α-Actinin (Sigma-Aldrich, Missouri, United States), Connexin 43/GJA1 (Abcam, Cambridge, United Kingdom) and were incubated overnight at 4°C. The primary antibodies were labeled with corresponding fluorescently conjugated secondary antibodies. Cell nuclei were stained with 4′,6-diamidino-2-phenylindole (DAPI) (Beyotime Biotechnology, Shanghai, China). The examination was done under Leica TCS SP8 laser confocal microscopic system (Leica Microsystems, Wetzlar, Germany).

### 2.8 Fluorescence-activated cell sorting (FACS)

After being digested and collected, the cells were fixed and permeabilized by Foxp3/Transcription Factor Staining Buffer Set (Invitrogen, Carlsbad, United States). Cells were incubated with Cardiac Troponin T antibody (Proteintech, Chicago, United States) on ice for 30 min to detect cardiomyocytes. Corresponding fluorescently conjugated secondary antibodies were used for flow cytometry analysis. The analysis was performed on a BD FACSCantoTM II flow cytometer (BioLegend, California, United States).

### 2.9 Transmission electron microscope (TEM)

We used TEM to study the microstructure of hiPSC-CMs. The cardiomyocytes differentiated to the 25th day were fixed with 2.5% glutaraldehyde at 4°C for 2 h. Then cells were post-fixed in 1% osmium tetroxide at 4°C for 2 h. The cells were then dehydrated by a graded ethanol series (30%-50%-70%-80%-95%-100%-100%). During dehydration, we used 3% uranyl acetate in 70% ethanol to stain the cells. After dehydration, cells were embedded in Epon 812. Ultrathin sections (700 nm) were cut with a ultramicrotome (LEICA, EM UC7, AT) and stained with lead citrate for further recording and analysis by electron microscopy (HITACHI, H-7650, JP).

### 2.10 Patch clamp

To record the cellular action potentials, hiPSC-CMs were reseeded on a glass bottom Petri dish (801002, NEST, CN). An inverted microscope (Ti−U, Nikon, JP) equipped with a patch clamp amplifier (MultiClamp700B, Axon CNS, United States) was used to record the cellular action potentials. Whole-cell patch clamp was performed on the cells at 36°C–37°C using a sharp microelectrode with a tip resistance between 3 MΩ and 5 MΩ which was made by pulling a standard-wall borosilicate glass capillary tube (B15023F, VitalSense, CN) with a micropipette puller (P97, Shutter Instrument, United States). The action potential inner solution was composed of 2.0 mM MgCl2, 150 mM KCl, 2.0 mM Na2ATP, 5.0 mM EGTA and 10 mM HEPES, and pH was adjusted to 7.2 with Tris. The action potential outer solution was composed of 1.0 mM MgCl2, 2.0 mM CaCl2, 5.0 mM KCl, 130 mM NaCl, 10 mM sucrose, 10 mM HEPES and 20 mM glucose, and pH was adjusted to 7.4 with Tris.

### 2.11 qPCR

The expression of myocardial maturation related genes was investigated by reverse transcription-polymerase chain reaction. On day 15 of the differentiation protocol, the cells were collected and resuspended in Trizol (ThermoFisher, Waltham, United States) to extract total RNA. The quantity and purity of RNA were validated using Nanodrop spectrophotometer (ThermoFisher, Waltham, United States). RNA was extracted by isopropanol and reverse transcribed into cDNA by PrimeScript RT reagent Kit (TaKaRa, totokyo, Japan). The PCR assay was conducted by QuantiNova SYBR Green PCR Kit (QIAGEN, Frankfurt, Germany) and performed on the CFX Connect Real-Time System (Bio-Rad, California, United States) for 2 min at 95°C, followed by 33 cycles (95°C for 5 s, 65°C for 60 s). The expression of target genes was normalized against 18 S and processed using the 2^dCt method. The primer sequences used were as follow.

**Table:** 


18S-F	GTA​ACC​CGT​TGA​ACC​CCA​TT
18S-R	CCA​TCC​AAT​CGG​TAG​TAG​CG
CTNT-F	ATG​AGC​GGG​AGA​AGG​AGC​GGC​AGA​AC
CTNT-R	TCA​ATG​GCC​AGC​ACC​TTC​CTC​CTC​TC
MYH6-F	ACA​GTC​ACC​GTC​TTC​CCA​TTC
MYH6-R	ACA​GTC​ACC​GTC​TTC​CCA​TTC
MYH7-F	ACC​AAC​CTG​TCC​AAG​TTC​CG
MHY7-R	TTC​AAG​CCC​TTC​GTG​CCA​AT
CX43(GJA1)-F	GGT​GAC​TGG​AGC​GCC​TTA​G
CX43(GJA1)-R	GCG​CAC​ATG​AGA​GAT​TGG​GA

### 2.12 Calcium imaging

hiPSCs-CMs were incubated with 5 mM rhod2 (R14220, LIFE TECHNOLOGIES, United States) for 60 min at 37°C. Prior to fluorescence measurements, cells were washed in indicator-free 1,640, and then incubated for a further 30 min. Afterwards, spontaneous increases in intracellular Ca2+ concentration was imaged using a fluorescent microscope (Leica, Germany) at 561 nm wavelength. Substrates were connected to a pacemaker (YC-3 Bipolar Programmable Electrical Stimulator, CHINA) and paced 2 ms, 5 V/cm, 1 Hz. The experiment was repeated 3 times, where each experiment was recorded for at least 1 minute. Data were analyzed using Leica Application Suite X 3.5.6.

### 2.13 Synchronous pacing assessment

C57BL/6 mice (6 weeks old) were purchased from JSJ-LAB, Shanghai, China. The Animal Care and Experiment Committee of the Shanghai Children’s Medical Center approved the experimental protocols (Approval No, SCMC-LAWEC-2021-011). Mice were anesthetized with isoflurane and were maintained on isoflurane inhalation. The hearts were excised and perfused with Tyrode’s solution, then arranged on aligned electrospun rGO/PLCL membranes or aligned electrospun PLCL membranes. Substrates were connected to a pacemaker system (ALC-EPS, CN) and paced 2 ms, 2 V/cm, 1 Hz. Electric signals were simultaneously recorded from these isolated hearts using Biological Signal Analysis System (ALC-MPA, CN.) 18 mice were randomly divided into 3 groups (*n* = 3 or 12 per group). 1 heart arranged on aligned electrospun rGO/PLCL membranes; 1 heart arranged on aligned electrospun PLCL membranes; 4 hearts arranged on aligned electrospun rGO/PLCL membranes. The experiment was repeated 3 times. Data were analyzed using ALC-MPA software.

### 2.14 Statistical analysis

All data presented are expressed as mean ± standard deviation. Statistical analysis was carried out using single-factor analysis of variance. A value of *p* < 0.05 was considered statistically significant.

## 3 Results

### 3.1 Preparation and characterization of rGO/PLCL membranes

**FIGURE 2:**
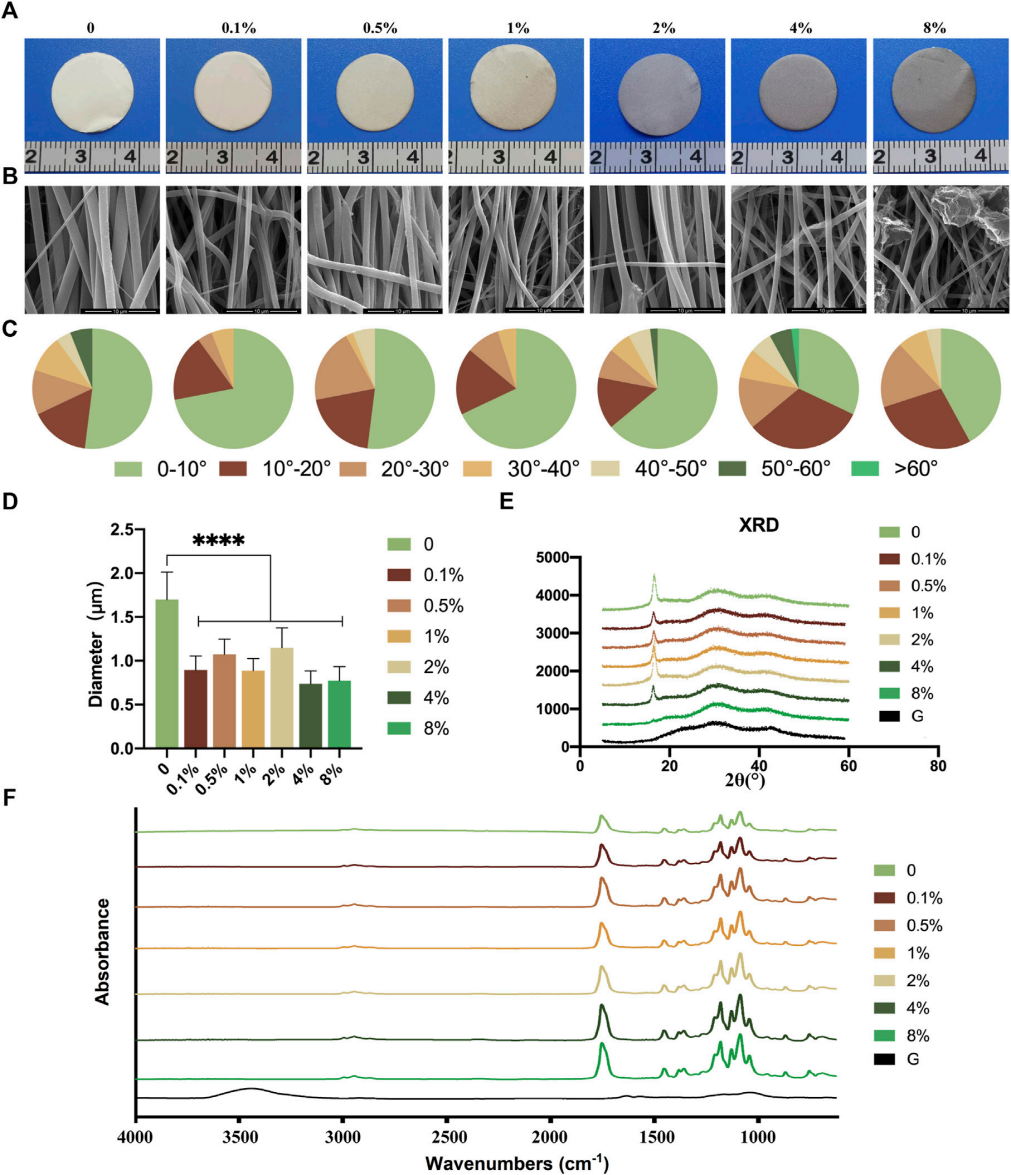
Fabrication and structure of the electrospun rGO/PLCL scaffolds. **(A)** Gross view of the electrospun rGO/PLCL scaffolds; **(B)** SEM images of the electrospun rGO/PLCL scaffolds, scale bar: 10 μm; **(C)** Angular distribution of the electrospun rGO/PLCL scaffold fibers; *n* = 200 per group. **(D)** Diameter of the electrospun rGO/PLCL scaffolds; *n* = 200 per group. **(E)** XRD pattern of rGO/PLCL scaffolds. **(F)** FTIR spectra of the electrospun rGO/PLCL scaffolds.

rGO/PLCL membranes with varying concentrations were prepared through electrospinning a mixed solution of rGO and PLCL. rGO was evenly distributed in the solution and the color of the solution gradually turned black as the rGO concentration increased (Supplementary Figure S1A). As rGO concentration increased, the membranes produced through electrospinning became gradually darker (Figure 2A). The morphological fabrication of these membranes appeared uniform and smoothly aligned, as obtained through scanning electron microscopy (Figure 2B). We next analyzed the arrangement of the fibers in the membranes at varying concentrations (Figure 2C). The fiber diameter of PLCL without rGO was 1.6986 ± 0.3133 μm, which was significantly larger than that of the fiber containing rGO of different concentrations (from 0.7386 ± 0.1447 μm to 1.1484 ± 0.2272 μm) (Figure 2D). The angle analysis indicated that 90% of the fibers were distributed within 40°. Among them, the proportion of the group without rGO and the low concentration rGO group (0.1, 0.5, 1, and 2 wt%) in the range of 0–10° were 52, 72, 52, 68, and 64% respectively. When the concentration of rGO reached 4%, the filaments distributed in the range of 0–10° decreased to 32% and the parallelism decreased significantly. When the concentration reached 8%, the filament morphology changed and the presence of more rGO was evident in the sample, which was caused by the unstable Taylor cone.

Fourier transform infrared spectroscopy (FTIR) is commonly used to determine the surface structure of electrospinning membranes. The persistently large peak observed at 3,448 cm−1 in the FTIR spectrum of rGO was mostly due to the moisture contained in the κBr pellets, which could not be avoided (Figure 2F) (Shin et al., 2016). The characteristic ester (C=O) absorption peak of PLCL was observed at 1755 cm−1, and the C–O–C stretching vibrations were observed at 1,088, 1,130, and 1,182 cm−1 (Figure 2F), which is in agreement with previous reports (Suhaeri et al., 2017; Rahmani et al., 2019). The rGO spectrum exhibited only slight absorption peaks. In the rGO/PLCL membranes, no new peaks were detected when rGO was adsorbed onto the fibers, demonstrating that rGO was fully exfoliated and homogeneously distributed in the polymer matrix. The characteristic absorption peaks of PLCL decreased as the concentration of rGO increased. Similar results were obtained in the X-ray diffraction analysis, as shown in Figure 2E. The contact angles of the membranes were measured (Supplementary Figures S1B, C). It was observed that there was no significant variance in the contact angle between the membranes with rGO and the membranes without rGO. Therefore, the addition of rGO does not appear to affect the hydrophilicity of membranes.

### 3.2 Mechanical properties of the rGO/PLCL membranes

**FIGURE 3:**
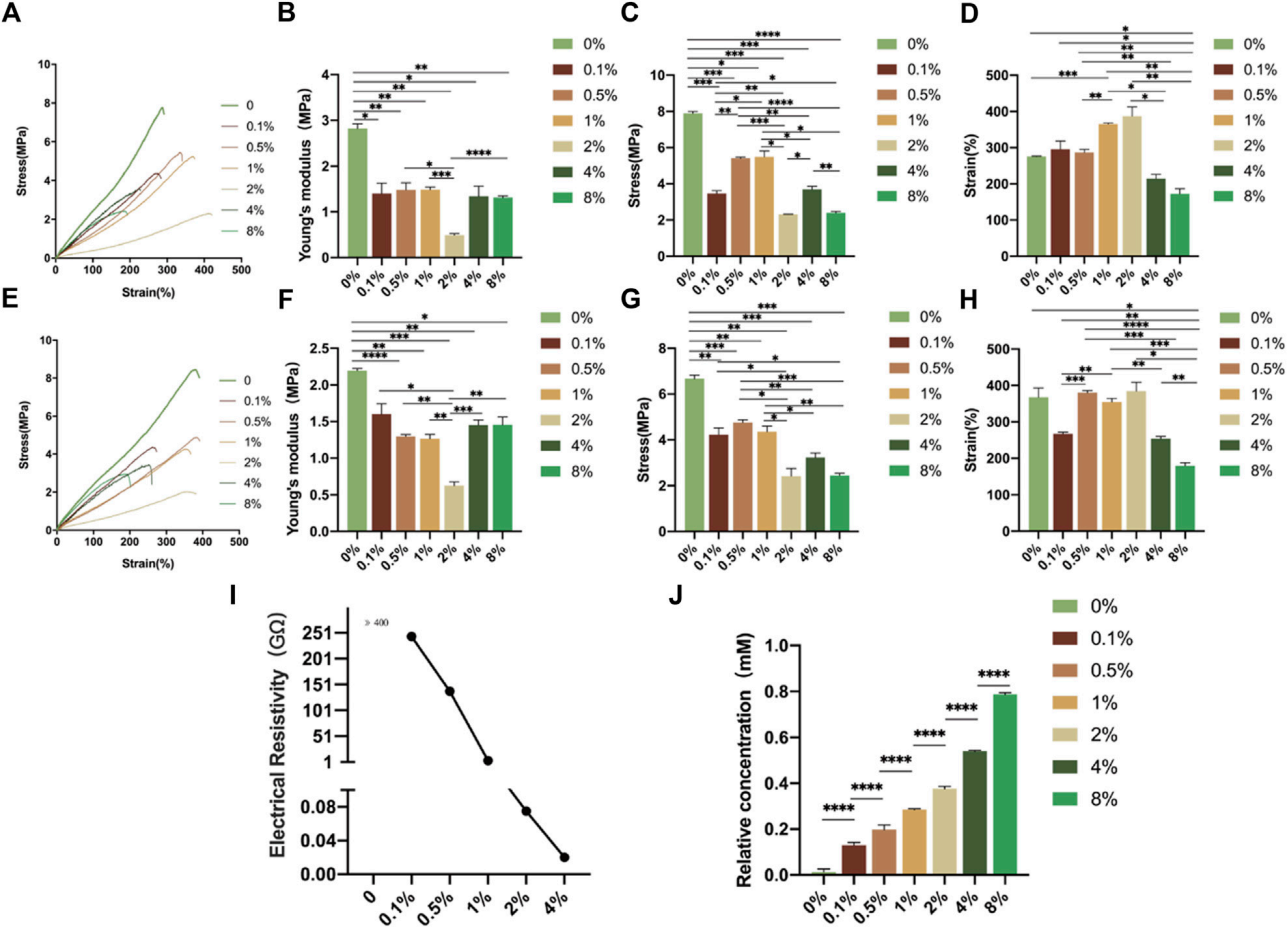
Characterization of the electrospun rGO/PLCL scaffolds. **(A)** Stress-strain curve of electrospun ordered rGO/PLCL under dry conditions; **(B–D)** Comparison of elastic modulus, **(B)** breaking strength **(C)** and maximum tensile strength **(D)** degree of electrospun ordered rGO/PLCL under dry conditions. Data were expressed as mean ± SD, *n* = 3 per group; **(E)** Stress-strain curve of electrospun ordered rGO/PLCL under wet conditions; **(F–H)** Comparison of elastic modulus **(F)**, breaking strength **(G)** and maximum tensile degree **(H)** of electrospun ordered rGO/PLCL under wet conditions. Data expressed as mean ± SD, *n* = 3 per group; **(I)** Electrical conductivity of the electrospun rGO/PLCL scaffolds; **(J)** The relative antioxidant capacity of the electrospun rGO/PLCL scaffolds. *n* = 5 per group.

The tensile strength data of the different membranes are presented in Figures 3A–H. The membranes with rGO have lower Young’s modulus (dry: from 0.49 ± 0.03 to 1.48 ± 0.06 MPa, hydrated: from 0.63 ± 0.05 to 2.20 ± 0.03 MPa) than the membranes without rGO (dry: 2.82 ± 0.10 MPa, hydrated:2.20 ± 0.03 MPa) (Figures 3B, F), which revealed that the addition of rGO softened the membranes. At 2 wt% rGO, Young’s modulus decreased to 0.49 ± 0.03 MPa (dry). However, the strains at rupture of dry 2 wt% rGO membranes exceeded 387.2% ± 25.6%, and that of wet membranes was 285.3% ± 24.3% (Figures 3D, H). During the whole process, the volume of the membranes did not change significantly, so the above results indicated that the membranes are highly elastic. Previous studies have shown that the stiffness of heart muscle is 10–20 kPa at the beginning cycle of diastole, and 200–500 kPa at the end of diastole (Chen et al., 2008). The heart muscle patches were therefore designed to have a stiffness in the range of several tens kPa to 1 Mpa (Chen et al., 2008; Huang et al., 2021). The stress-strain curve and the statistical results of stress of different membranes demonstrated that the mechanical properties of all the membranes were different from those of natural myocardium (Figures 3A, C, E, G). However, the mechanical feature of 2 wt% rGO was the best choice considering both stress-strain curve and Young’s modulus.

### 3.3 Electrical conductivity and antioxidant activity of the rGO/PLCL membranes

To measure the conductivity of rGO, we measured the resistivity of the rGO membranes with varying concentrations. The electrical resistivities of the membranes are shown in Figure 3I. The resistivity of the membranes without rGO was significantly higher than 400 Ω. Note that the membranes with 8 wt% rGO cannot obtain accurate information on their electrical resistivity owing to the uneven distribution of rGO (data not shown). The resistivity decreased in a concentration-dependent manner and when the rGO concentration reached 2 wt%, the resistance of the membranes decreased significantly, with an electrical resistivity of 75 MΩ. Therefore, an internal network was obtained, which increased the electrical conductivity of the membrane.

The antioxidant activities of rGO materials at different concentrations were tested using the ABTS method. The ABTS free radical scavenging activity was improved by adding rGO to the membranes (Figure 3J). The relative antioxidant activity increased nearly 50 fold in the 8 wt% and nearly 30 fold in the 2 wt% rGO/PLCL group compared to the group without rGO. Therefore, rGO membranes can effectively resist reactive oxygen species, which is extremely beneficial for the survival of myocardial cells on membranes (Dhalla et al., 2022).

### 3.4 Biocompatibility of the rGO/PLCL membranes *in vitro*

**FIGURE 4:**
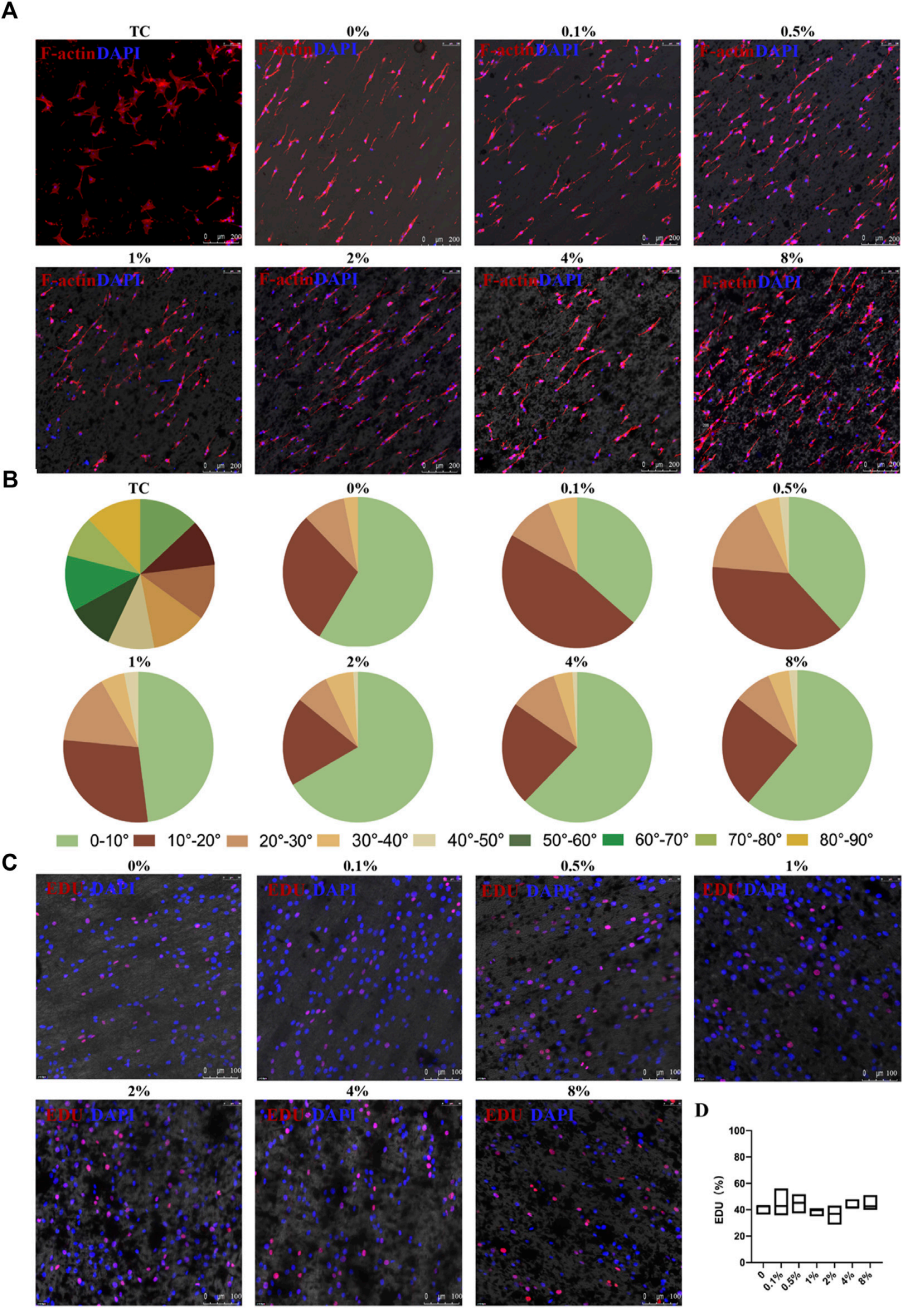
Cellular arrangement and proliferation of 3T3 cells on the aligned rGO/PLCL electrospun scaffolds. **(A)** 3T3 cell arrangement on the electrospun rGO/PLCL scaffolds or treated tissue culture dishes, scale bar: 200 μm; Blue, DAPI for nucleic acid staining. Red, phalloidin, fluorescent staining of cell structure; **(B)** Angular distribution of the 3T3 cell cultured on the electrospun rGO/PLCL scaffolds and on treated tissue culture dishes. *n* = 200 per group; **(C)** 3T3 cell proliferation on the electrospun rGO/PLCL scaffolds, scale bar: 100 μm; Blue, DAPI for nucleic acid staining. Red, EDU, DNA replication reactive dyes. Scale bars: 100 μm; **(D)** Statistical analysis of EDU proliferation assay on the aligned rGO/PLCL electrospun scaffolds.

The membranes were designed to orient the cell direction using anisotropic topography. To explore the effect of these membranes on cell arrangement, 3T3 fibroblast cells were seeded on membranes containing varying concentrations of rGO and 24 h later the cytoskeleton was stained after the cells completely adhered to the membranes using phalloidin, as shown in Figure 4A. It was observed that the long axis of the cells was consistent with the arrangement of the fibers in the membranes. Through the statistical analysis of the angle between the long axis of the cells and the long axis of the membrane fibers (Figure 4B), we observed that more than 90% of the cells were within 30°. The cells within 10°, 2%, 4%, and 8% rGO membranes were more than 60%, while the cells cultured on the ordinary cell culture dish showed a disordered distribution. These results demonstrated that the membranes had a certain guiding effect on 3T3 cells, which could be useful for simulating the directional arrangement of natural cardiomyocytes.

However, the toxicity of rGO remains controversial. Some previous studies have suggested that rGO exhibits dose-dependent toxicity (Zhang et al., 2011; Xu et al., 2015). To test whether rGO exhibits cell toxicity in our system, we inoculated 3T3 cells onto the membranes and measured their proliferation using an EDU proliferation kit. The correlation between cell proliferation and cell health has previously been reported (Anjum et al., 2022). The results demonstrated that the proliferation of 3T3 cells without rGO was 40.84% ± 3.81%, whereas the proliferation ranged from 36.24% ± 7.25% to 44.88% ± 10.28% for the membranes with rGO (Figure 4C). Interestingly, there was no statistical significance in the variance of proliferation among the 3T3 cells in varying rGO percentages (Figure 4D). In fact, the proliferation potential of the 3T3 cells was neither negatively or positively affected by rGO, which is consistent with some previous reports (Rehman et al., 2019; Wang et al., 2019; Yao et al., 2023), as such we concluded that rGO used at these concentrations did not induce cellular toxicity.

### 3.5 Differentiation and characterization of the hiPSC-CMs

**FIGURE 5:**
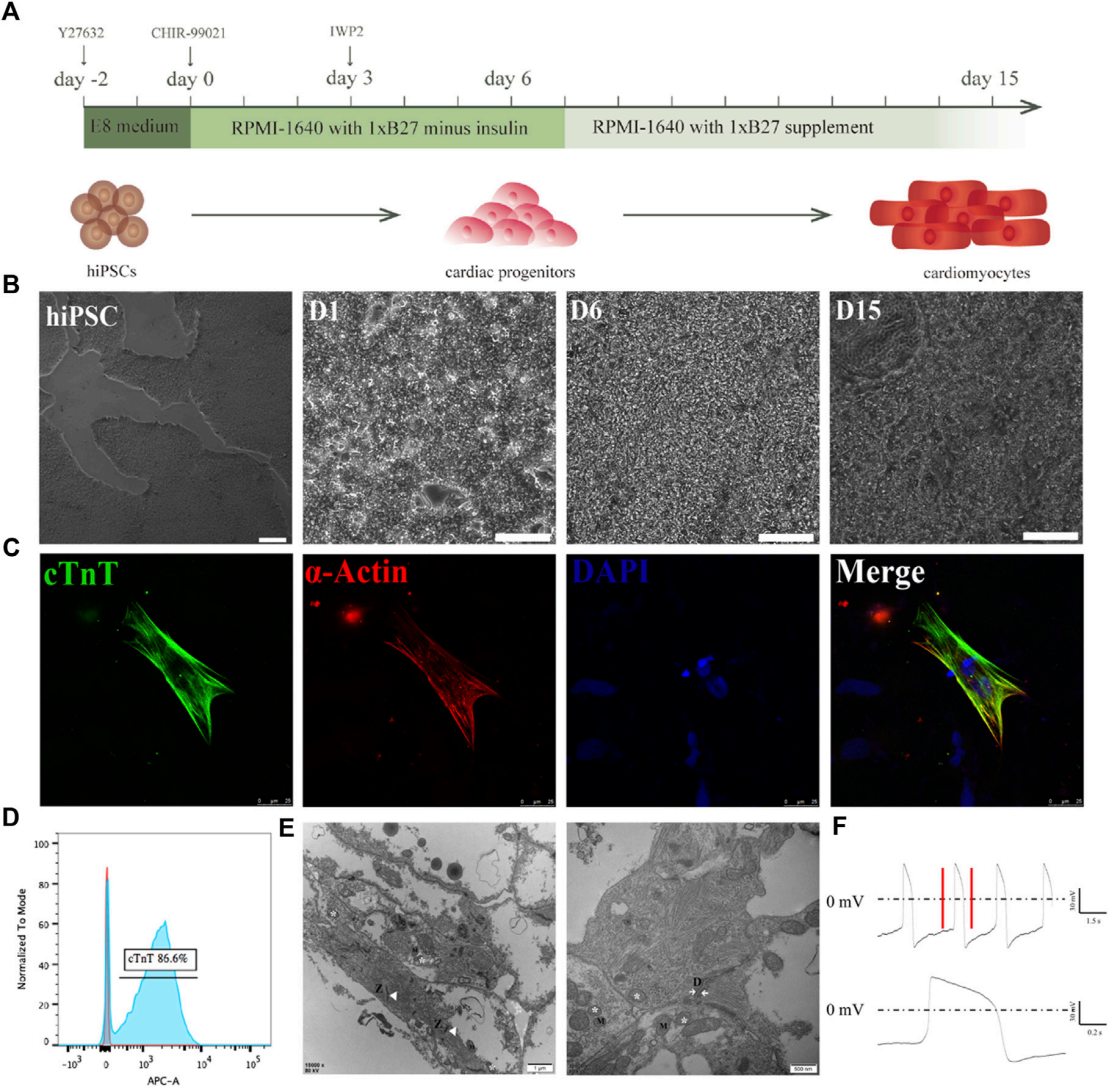
Differentiation of cardiomyocytes from iPSCs. **(A)** Schematic of the cardiomyocyte differentiation protocol; **(B)** The morphological changes of cells during cardiomyocyte differentiation. Scale bar: 100 μm; **(C)** immunofluorescence staining of the cardiomyocytes. Green, cTnT; Red, α-actin; Blue, DAPI. Scale bar: 25 μm; **(D)** Flow cytometry of cTnT^+^ cells; **(E)** SEM image of the hiPSC-CMs, scale bar 500 nm and 1 μm. Z line (Z), mitochondrion (M), Moistening plate (N); **(F)** Typical action potential of an individual hiPSC-derived cardiomyocyte recorded via patch clamp.

With an interest to cultivate hiPSC-CMs on electrospun rGO- treated membranes, we employed a previously published method to differentiate hiPSCs into CMs by modulating Wnt signaling using small molecules (Figures 5A–C) (Rahmani et al., 2019). Prior to the cardiac differentiations of the hiPSCs, we confirmed the homogenous nature of the cells by staining pluripotency markers, namely SOX2, TRA-1-60, NANOG, and OCT4 (Supplementary Figures S2A–D). On the 10th day of differentiation, spontaneously beating CMs were visible (Movie.1). The synchronously beating myocardial cells presented with sarcomeric protein structures (Figure 5C). On the 15th day of differentiation, flow cytometry analysis demonstrated that more than 80% of the cells in culture expressed the myocardial marker cTnT (Figure 5D). A clear Z-line, as well as sarcomere and mitochondrial structures were observed in differentiated hiPSC-derived myocardium (Figure 5E). Furthermore, action potential recordings of the spontaneously beating CMs appeared ventricular-like as denoted by patch clamp recordings, which corresponds with the classification criteria of hiPSC-CMs as previously reported (Figure 5F). These data confirm the robustness and consistency of previously published protocols for producing high quality hiPSC-CMs, to be tested on the rGO/PLCL membranes.

### 3.6 Engineering a patch with rGO/PLCL membranes and hiPSC-CMs

**FIGURE 6:**
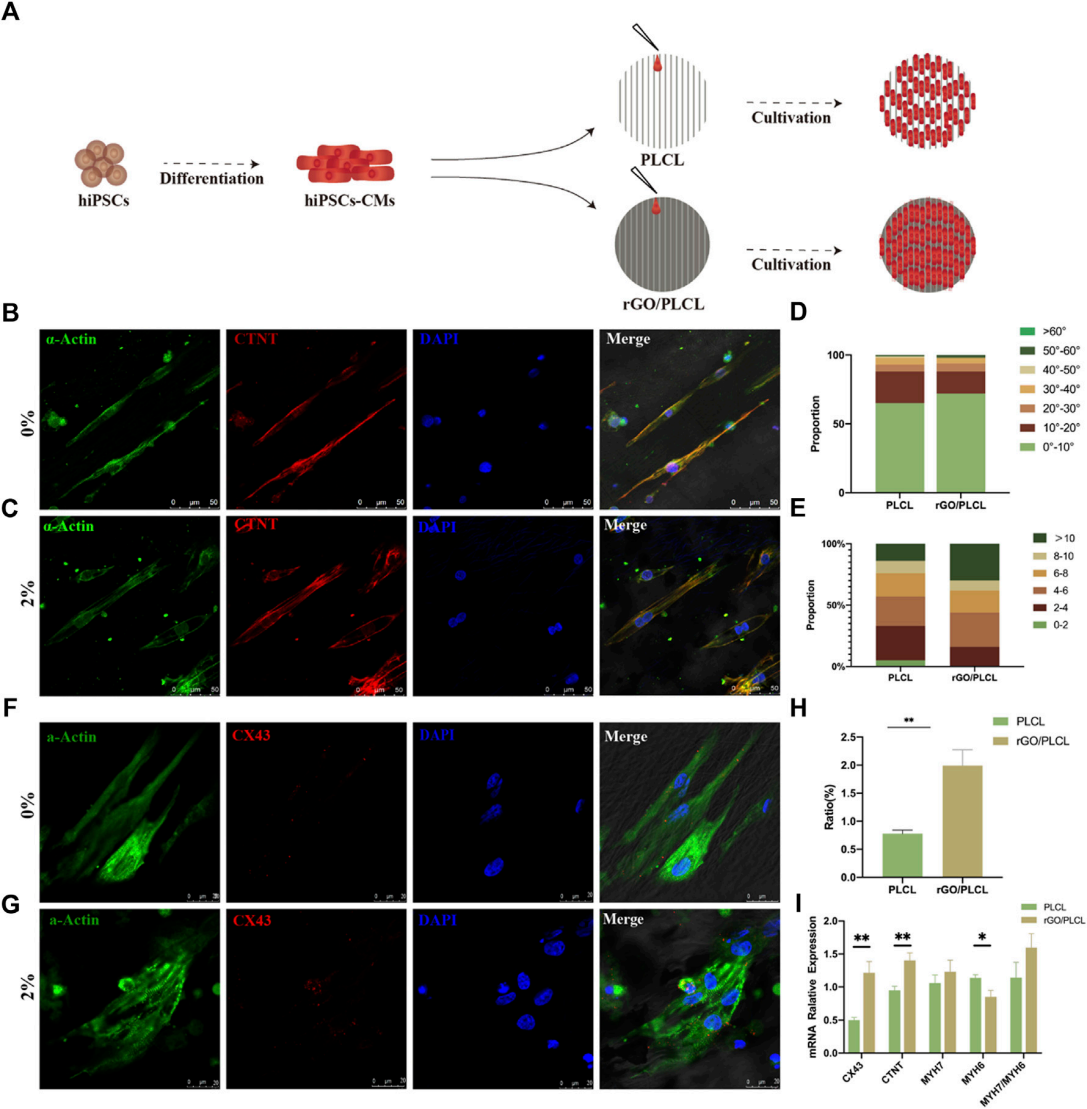
Cardiomyocyte alignment and connectivity on the electrospun rGO/PLCL scaffolds. **(A)** Pattern of the cardiomyocytes on scaffolds containing PLCL and 2% w/w rGO/PLCL; **(B,C)** Arrangement of cardiomyocytes on the PLCL scaffolds **(B)** and 2% w/w rGO/PLCL scaffolds, **(C)** Green, α-actin; Red, cTnT; Blue, DAPI. Scale bar: 50 μm; **(D)** Angular distribution of the cardiomyocyte cultured on PLCL scaffolds and 2% w/w rGO/PLCL scaffolds, *n* = 200 per group; **(E)** Aspect ratio of cardiomyocytes cultured on the PLCL scaffolds and 2% w/w rGO/PLCL scaffolds. *n* = 200 per group; **(F,G)** Representative photomicrographs of immunostained CMs on the scaffolds containing either PLCL **(F)** or 2% w/w rGO/PLCL. **(G)** Green, α-actin; Red, CX43; Blue, DAPI. scale bar: 20 μm; **(H)** Bar graph representing levels of Cx43 expression in CMs cultivated on either PLCL or rGO/PLCL scaffolds; **(I)** Gene expression profiles of well-known cardiac genes from CMs cultured on PLCL sand 2% w/w rGO/PLCL scaffolds. CX43, connexin 43; cTnT, cardiac troponin-T; MHY7, myosin heavy chain 7; MHY6, myosin heavy chain 6.

To determine the effect of the electrospun rGO/PLCL membranes on hiPSC-CMs, we selected the 2 wt% rGO membranes as an experimental group and employed the membranes without rGO as a control. iPSC-CMs at day 15 of the differentiation protocol were seeded on the membranes and the morphology of the cells were analyzed 24 h later (Figure 6A). We found that the CMs on the two materials could be arranged in an orderly fashion (Figures 6B, C). The proportion of CMs within 10° was 65% (PLCL) and 72% (rGO/PLCL), respectively. Approximately 90% of the cells were within 30° of each other (Figure 6D), which substantiates the guiding effects of CMs on both PLCL and rGO/PLCL. The cells’ length to width ratio was also analyzed. The membranes containing 2 wt% rGO had a larger aspect ratio. 30% of the cells on rGO/PLCL contained an aspect ratio greater than 10, much larger than the proportion of the cells on PLCL (Figure 6E). Sarcomeric striation is a unique phenotype of CMs and was more visible in CMs seeded on the rGO membranes than CMs on the PLCL membranes, which was made evident by α-actinin staining and analysis (Figures 6F, G). Previous studies have shown that CMs with larger aspect ratio exhibit better contractility and higher maturity (Lundy et al., 2013; Suhaeri et al., 2017). In addition, connexins are typically observed in the heart at the intercalated disks of adjacent CMs, where they facilitate electrical current flow that coordinates CM contraction to sustain pump function (Schulz et al., 2015). Interestingly, we found that the CMs cultivated on membranes containing rGO more significantly promoted the expression of Cx43 (1.99% ± 0.28%) than those seeded on membranes without rGO (0.78% ± 0.06%) (Figure 6H). To elucidate the influence of rGO on CM gene expression, we analyzed the expression patterns of several well-known cardiac muscle genes using real-time qPCR. Intriguingly, the gene expression patterns of both Cx43 and the CM-specific myofilament marker cTnT were significantly higher in CMs cultured in 2 wt% rGO membranes than that of CMs cultivated on membranes without rGO (Figure 6I). Further, the ratio of MYH7/MYH6, which is related to CM sarcomeric maturity, was significantly increased amongst the CMs cultivated on rGO membranes (Figure 6I). These findings suggest that cultivating CMs on rGO-containing membranes enhances the connectivity and maturity of CMs.

### 3.7 Conduction-consistent characteristic of the cardiac patch

**FIGURE 7:**
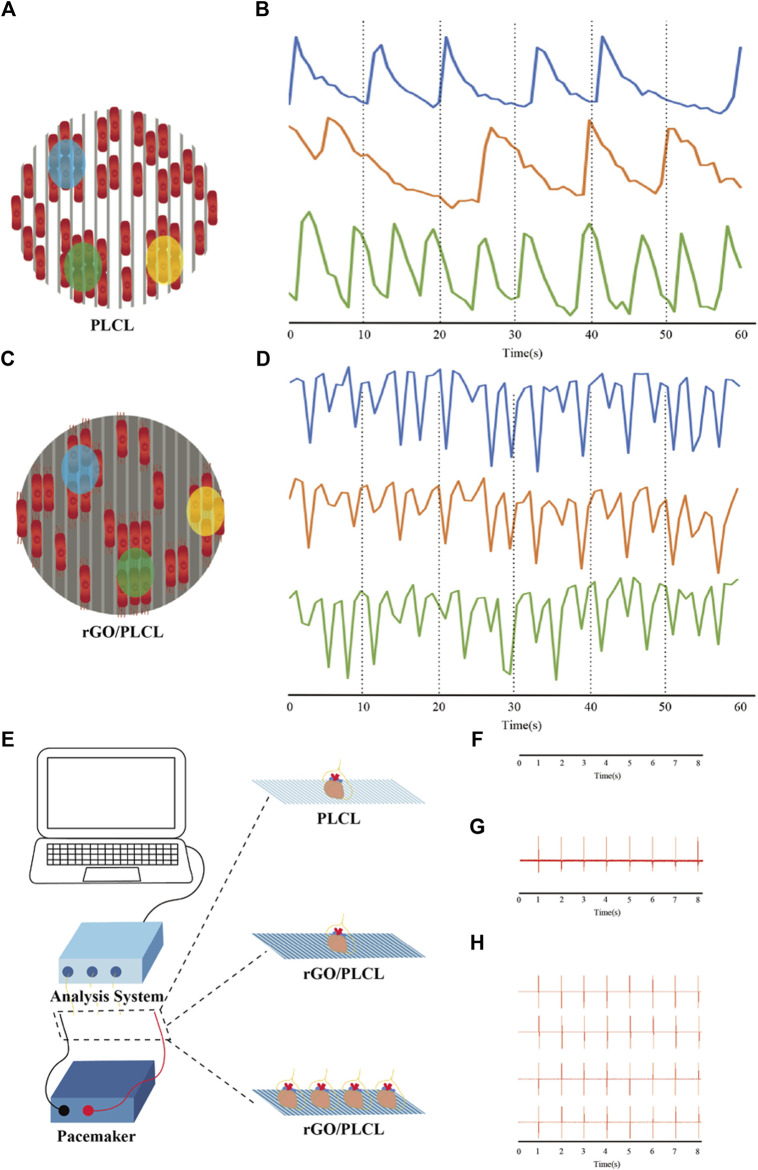
Intracellular Ca^2+^ transient and external electrical stimulation. **(A–D)** Schematic diagram and electrical tracings representing CMs cultured on PLCL **(A,B)** or rGO/PLCL **(C**,**D)** electrospun material in three locations; **(B)** the change of Ca^2+^ concentration of CMs cultured on PLCL electrospinning material in three isolated areas; **(D)** the change of Ca^2+^ concentration of CMs cultured on rGO/PLCL electrospnning material in three isolated areas; **(E)** Schematic drawing of electrical recordings of mouse heart(s); **(F)** Electrical signal tracking display of mouse heart on PLCL electrospinning material; **(G)** Electrical signal tracking display of mouse heart on rGO/PLCL electrospinning material; **(H)** Electrical signal tracking display of four isolated mouse hearts on rGO/PLCL electrospinning material. The electrical stimulation frequency was 1 Hz.

To explore the intercellular communication between CMs cultured on rGO/PLCL membranes, transient calcium (Ca^2+^) signaling and analysis was performed. Cells were first loaded with dyes, and thereafter films were captured from different sites of the PLCL and rGO/PLCL membranes under electrical stimulation (2 ms, 5 V, and 1 Hz). Three different random sites were selected and the increase in levels of Ca^2+^ ion concentration was carefully monitored, which was indicated by the fluorescent intensity of the dye. On PLCL membranes, intercellular Ca^2+^ occurred at different frequencies at all three consideration points (Figures 7A, B). However, CMs cultured on rGO/PLCL membranes exhibited synchronous Ca^2+^ peaks at the points considered (Figures 7C, D). The calcium transient is triggered by electrical signals, so we speculated that rGO/PLCL membranes promoted the consistent electrical signal changes between hiPSC-CMs at the cellular level.

Next, to detect the influence of rGO/PLCL membranes on electrical signal transmission at the tissue level, we carried out real-time electrical signal detection in isolated hearts. We placed different numbers of isolated mouse hearts on membranes connected to electrical stimulators and simultaneously monitored their electrical signals (Figure 7E; Supplementary Figures S3A–E). Continuous electrical tracking demonstrated that the electrical signals of a single isolated mouse heart on PLCL membranes could not be captured, even when the maximum stimulation voltage was 10 V (Figure 7F; Supplementary Figure S3F). In addition, we used an electrical stimulation device to evaluate the ability of rGO/PLCL membranes to accommodate external electrical stimulation. A single isolated mouse heart exhibited a regular electrical signal/rhythm on rGO/PLCL membranes, which was consistent with the signal output frequency of the electrical stimulator (2 ms, 2 V/cm, and 1 Hz) (Figure 7G; Supplementary Figure S3G). Succeeding this, we placed four isolated mouse hearts on rGO/PLCL membranes connected to an electrical stimulator and performed synchronous electrical stimulation. Intriguingly, the electrical signal tracking indicated that the four isolated mouse hearts exhibited synchronous electrical signals with a regular rhythm, which was consistent with the output frequency of the electrical stimulator (Figure 7H; Supplementary Figure S3H). Therefore, the rGO/PLCL membranes exhibited excellent synchronous electrical conductivity at both the cellular and tissue levels.

## 4 Discussion

Cardiovascular disease is the leading cause of death worldwide. The construction of engineered myocardial patches offer significant value in heart disease research including the screening of therapeutic drugs and alternative treatment options for cardiac injury. In the anatomical normal heart, CMs present with directional arrangement, a key feature enabling synchronous contractions of CMs. Therefore, the construction of a myocardial muscle patch that allows the directional arrangement of CMs and consistent conduction is critical.

Electrospinning is an emerging technology used in tissue engineering (Shi et al., 2022). The manufacturing methods of electrospinning are readily adjustable and can provide cell-specific surface structures of the membranes, it has been used in several studies to guide cell arrangement (Yuan et al., 2022). The results herein demonstrated that electrospun rGO/PLCL membranes effectively guided cell arrangement with excellent physicochemical properties and cell compatibility. Due to the rapid growth rate of 3T3 cells, extensive culture on the membranes was not feasible. As such we were not able to employ 3T3 to measure the long-time biological toxicity of rGO/PLCL membranes. Next, we used EDU to detect the proliferation of 3T3 on the membranes, in order to assess levels of biological toxicity of the rGO/PLCL membranes. Previous studies have shown minimal toxicity of rGO to different cell types by means of live/dead staining, MTT and other applications (Rehman et al., 2019; Wang et al., 2019; Yao et al., 2023). Taken together, we believe that rGO exhibits low biological toxicity and as such the use of electrospinning technology to prepare rGO/PLCL scaffolds for constructing myocardial patches is feasible.

Enhancing biologically engineered myocardial muscle patches so that they retain electrical conduction consistency remains a major hurdle. According to previous studies, researchers have used a variety of electroactive materials to enhance the effects of external signals and cell signal transduction, such as conductive polymers or oligomers (Gelmi et al., 2016), carbon nanotubes (Mooney et al., 2012), carbon nanofibers (Stout et al., 2011), and gold nanowires (Ravichandran et al., 2014). In this study, rGO was added to construct a myocardial muscle patch and provide better electrical conduction between CMs. The electrical conductivity of the membranes has been significantly improved, which was explained by the phenomena on the mobility of electrons through the nanoparticles. When nanoparticles are closed, several micro-tunnels are created; in this way, the electrons propagate or jump freely (Correa et al., 2019). Thereafter, we combined the membranes with hiPSC-CMs to construct the cardiac patch. CMs receive and respond to electrical impulses and transmit electrical impulses to neighboring cells (Morley and Vaidya, 2001). We used transient Ca^2+^ analysis to explore the intercellular communication between CMs cultured on these membranes, and the transient distribution of Ca^2+^ confirmed the enhancement of intercellular communication between CMs cultured on the conductive material (Figure 7). We speculate that the regular synchronous beating rhythm of CMs grown on rGO/PLCL membranes may be due to: I) rGO/PLCL membranes improving the coupling between cells and promoting the synchronous contraction of adjacent cells by increasing the expression of Cx43 (Figures 6F–I); II) enhancing the electrical conductivity between cells, such that all cells, including isolated cells, can be excited at the same time under electrical pulses. III) The conductivity of the electrical signal may be achieved through the rGO/PLCL membranes. The cells cultivated on the membranes were simultaneously stimulated by electrical signals, and then consistent calcium transients were observed. We also confirmed that the rGO/PLCL membranes exhibit excellent synchronous electrical conductivity at the tissue level, which can be predicted that compared with PLCL membranes, rGO/PLCL membranes can promote integration (electrically) with the natural myocardium (Figure 7).

Previous studies have shown that PLCL is a biodegradable material (Haim Zada et al., 2020), and rGO has also been widely used in various biological materials (Rehman et al., 2019). Therefore, we speculate that the rGO/PLCL membranes used in this study may present with good degradability, which could be beneficial for *in vivo* applications. As such, our followup study will aim to assess the long-term toxicity, biodegradability and overall therapeutic potential of transplanted rGO/PLCL membranes in rodent myocardial infarction models.

## 5 Conclusion

In this research, we describe an engineered and conduction-consistent cardiac patch by combining hiPSC-CMs with aligned electrospun rGO/PLCL membranes. The fibers on the surface of the scaffolds displayed an ordered arrangement, excellent mechanical properties, and oxidation resistance. These properties were shown to effectively guide the arrangement of 3T3 cells and hiPSC-CMs. Transient Ca^2+^ analysis demonstrated that the addition of rGO exhibited excellent synchronous electrical conductivity at the cellular and tissue levels and the addition of rGO is conducive to the expression of maturation-related genes. In conclusion, human iPSC-CM electrospun nanofibers demonstrate significant potential for applications related to muscle tissue engineering.

## Data Availability

The original contributions presented in the study are included in the article/Supplementary Material, further inquiries can be directed to the corresponding authors.
