# Supplementary material for: Engineering a conduction-consistent cardiac patch with rGO/PLCL electrospun nanofibrous membranes and human iPSC-derived cardiomyocytes
Source: Front Bioeng Biotechnol. 2023 Feb 8;11:1094397. doi: 10.3389/fbioe.2023.1094397 (PMC9944832; doi:10.3389/fbioe.2023.1094397)
Supplement: Supplementary file 1 [file DataSheet1.PDF]

Supplementary

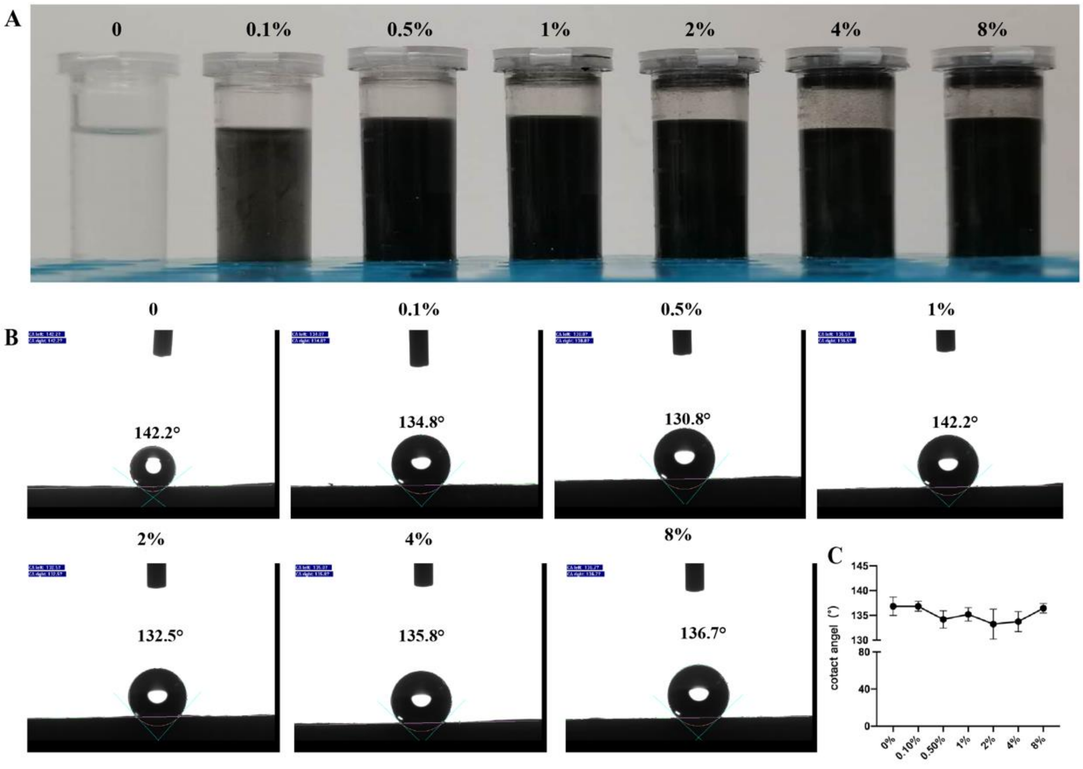

Figure S1 Fabrication and water contact angles of the aligned electrospun rGO/PLCL scaffolds

(A) the solution of the scaffold that mixed rGO with different concentrations and PLCL. (B) Water contact angles of the electrospun rGO/PLCL scaffolds. (C) Statistics of contact angle of the aligned electrospun rGO/PLCL scaffolds. n=3 per group.

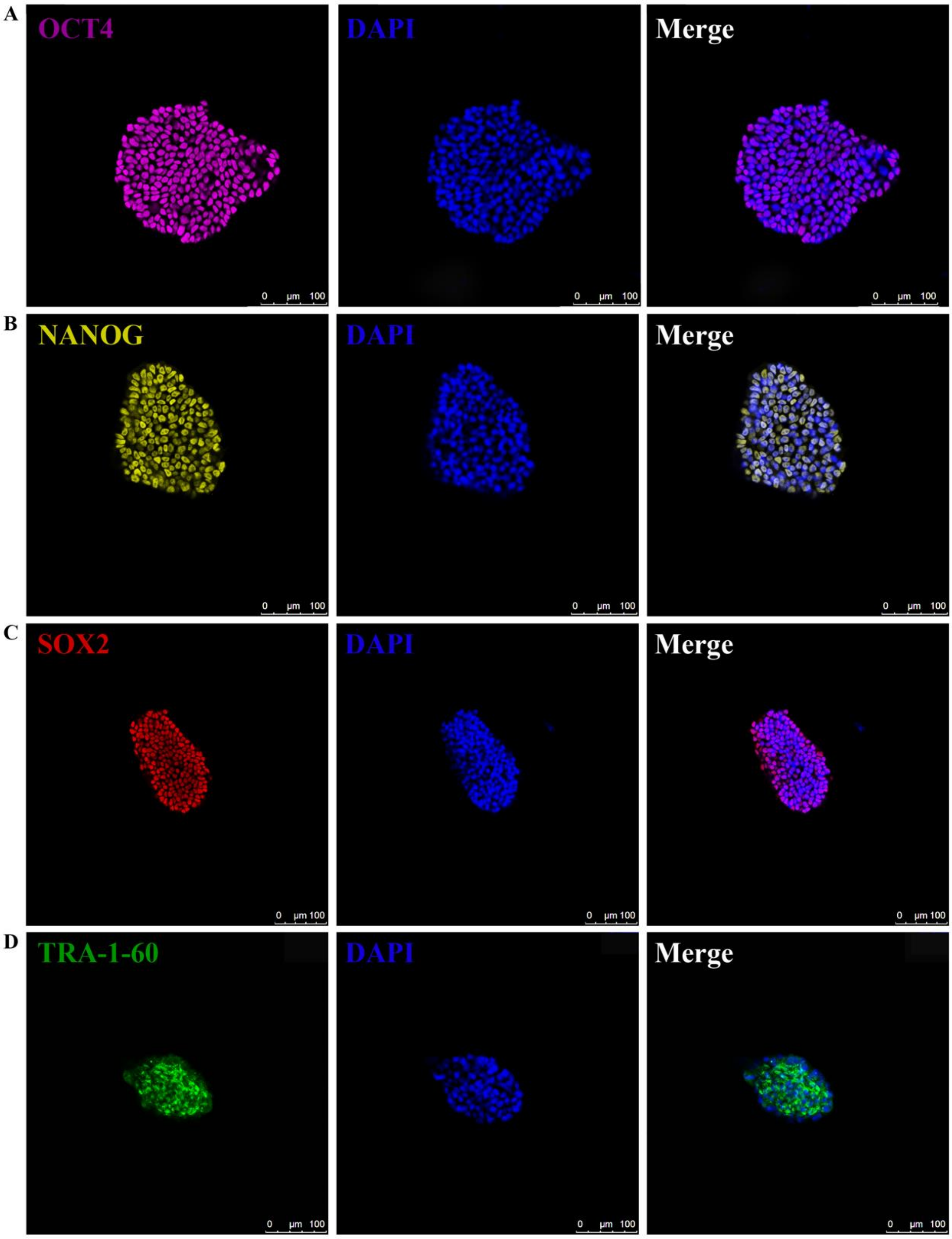

Figure S2 Expression of pluripotency markers visualized in hiPSCs

(A-D) Immunofluorescence staining of pluripotency markers OCT4, NANOG, SOX2, TRA-1-60. scale bar: 100 μm.

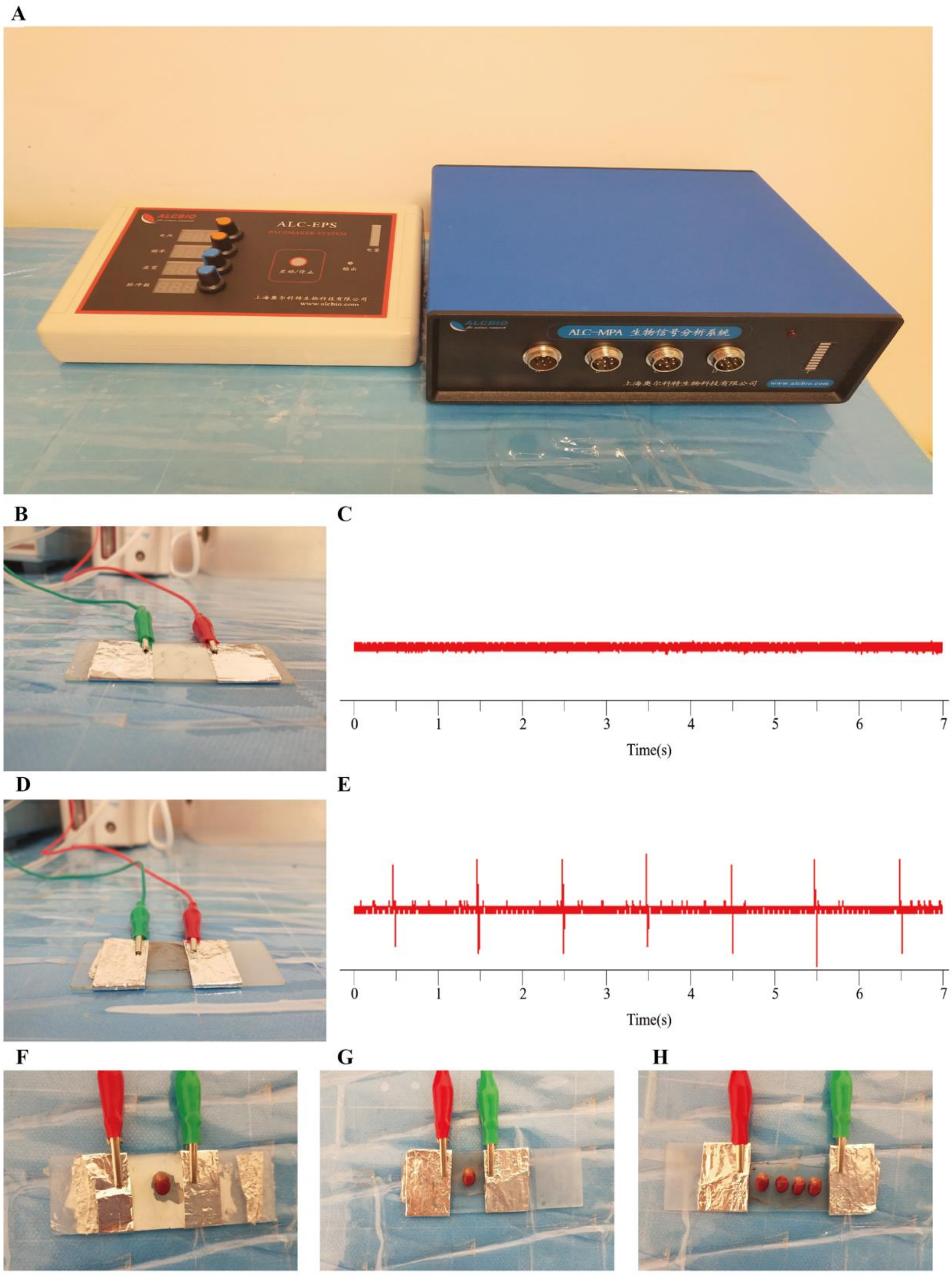

Figure S3 Assembly of a synchronous pacing system

(A) Left: pacemaker system (ALC-EPS, CHINA), right: Biological Signal Analysis System (ALC-MPA, CHINA). (B) PLCL scaffolds connected to the pacemaker. (C) Electric signals of PLCL scaffolds connected to the pacemaker. (D) rGO/PLCL scaffolds connected to the pacemaker. (E) Electric signals of rGO/PLCL scaffolds connected to the pacemaker. (F) A single heart on the PLCL scaffolds connected to the

pacemaker. (G) A single heart on the rGO/PLCL scaffolds connected to the pacemaker. (H) Four isolated hearts on the rGO/PLCL scaffolds connected to the pacemaker. The electrical stimulation frequency was 1 Hz.
